# The Clinical Significance of Myosteatosis in Survival Outcomes in Patients with Hepatocellular Carcinoma Treated with Sorafenib

**DOI:** 10.3390/cancers16020454

**Published:** 2024-01-20

**Authors:** Min Kyu Kang, Jeong Eun Song, Se Young Jang, Byung Seok Kim, Woo Jin Chung, Changhyeong Lee, Soo Young Park, Won Young Tak, Young Oh Kweon, Jae Seok Hwang, Byoung Kuk Jang, Yu Rim Lee, Jung Gil Park, on behalf of Daegu-Gyeongbuk Liver Study Group (DGLSG)

**Affiliations:** 1 Department of Internal Medicine, Yeungnam University Hospital, College of Medicine, Yeungnam University, Daegu 42415, Republic of Korea; kmggood111@naver.com; 2 Department of Internal Medicine, Daegu Catholic University Hospital, School of Medicine, Daegu Catholic University, Daegu 42415, Republic of Korea; ssong3004@naver.com (J.E.S.); kbs9225@cu.ac.kr (B.S.K.); chlee1@cu.ac.kr (C.L.); 3 Department of Internal Medicine, Kyungpook National University Hospital, School of Medicine, Kyungpook National University, Daegu 41944, Republic of Korea; magnolia1103@naver.com (S.Y.J.); psyoung0419@gmail.com (S.Y.P.); eworldcup@gmail.com (W.Y.T.); yokweon@knu.ac.kr (Y.O.K.); 4 Department of Internal Medicine, Keimyung University Dongsan Hospital, School of Medicine, Keimyung University, Daegu 42601, Republic of Korea; chung50@dsmc.or.kr (W.J.C.); gastro@dsmc.or.kr (J.S.H.); jangha106@dsmc.or.kr (B.K.J.)

**Keywords:** hepatocellular carcinoma, myosteatosis, sorafenib, survival

## Abstract

**Simple Summary:**

Research on the clinical impact of body composition parameters, such as myosteatosis, in patients with hepatocellular carcinoma (HCC) is limited. Our study examined the effects of computed tomography-based body composition measurements and traditional risk factors on the survival outcomes of sorafenib-treated HCC patients. Myosteatosis, evaluated using Hounsfield units, is associated with unfavorable survival outcomes, independent of traditional risk factors including vascular invasion, extrahepatic metastasis, and high alpha-fetoprotein levels. Two or more of these risk factors are associated with adverse outcomes. Thus, myosteatosis could serve as a prognostic factor in sorafenib-treated HCC patients.

**Abstract:**

The role of body composition parameters in sorafenib-treated hepatocellular carcinoma (HCC) patients is still not fully elucidated. Here, we aimed to evaluate the impact of computed tomography (CT)-based body composition parameters on the survival of such patients. In this multicenter study, we analyzed the data of 245 sorafenib-treated HCC patients from January 2008 to December 2019. Sarcopenia, visceral obesity, and myosteatosis were defined by using cross-sectional CT images at the third lumbar vertebra level. The effects of these parameters on overall survival (OS) and progression-free survival (PFS) were evaluated. The median age was 67.0 years (interquartile range: 61.0–78.0 year), and 211 patients (86.1%) were male. The median OS and PFS were 7.9 months and 4.8 months, respectively. Vascular invasion (hazard ratio (HR), 1.727; 95% confidence interval (CI), 1.258–2.371; *p* = 0.001), extrahepatic metastasis (HR, 1.401; 95% CI, 1.028–1.908; *p* = 0.033), alpha-fetoprotein level > 200 ng/mL (HR, 1.559; 95% CI, 1.105–2.201; *p* = 0.012), and myosteatosis (HR, 1.814; 95% CI, 1.112–2.960; *p* = 0.017) were associated with OS. Patient mortality was significantly higher in the group with two or more risk factors than in the group with fewer risk factors. In conclusion, myosteatosis may be a novel prognostic CT-based radiological biomarker in sorafenib-treated HCC patients.

## 1. Introduction

Hepatocellular carcinoma (HCC) has been reported to have the third-highest cancer-related mortality and sixth-highest cancer incidence worldwide [1]. HCC prognosis is classified according to the tumor characteristics, performance status, and liver function, including that classified using the Barcelona Clinic Liver Cancer (BCLC) staging system [2]. Patients with BCLC-0/A stage HCC have a median overall survival (OS) > 5 years, whereas those with BCLC-C stage HCC have a poorer prognosis, with median OS < 2 years [3].

Sorafenib (Nexavar; Bayer HealthCare, Leverkusen, Germany), an oral multi-tyrosine kinase inhibitor (TKI), has been widely used as a first-line systemic treatment for BCLC-C stage HCC classified as Child–Turcotte–Pugh (CTP) A or B, favorable performance status 1 or 2, portal vein thrombosis, and extrahepatic metastasis [2]. Despite the advent of atezolizumab and bevacizumab, sorafenib remains the first-line systemic therapy in cases with contraindications to atezolizumab and bevacizumab [3]. Given that the OS for sorafenib-treated HCC patients was 15.6–20.1 months for those with BCLC-B stage and 8.4–13.6 months for those with BCLC-C stage in real-world data, it is necessary to discover a novel prognostic factor and establish a risk model for individualized treatment [4,5]. Previous studies on prognostic factors for mortality in sorafenib-treated HCC patients have focused on tumor-associated factors, including extrahepatic metastasis, vascular invasion, high alpha-fetoprotein levels, and unfavorable liver function [6,7,8].

Computed tomography (CT)-based body composition parameters, including skeletal muscle mass, visceral adipose tissue (VAT), and subcutaneous adipose tissue (SAT), are emerging as potential prognostic factors for HCC treated with sorafenib [8,9,10,11,12]. Using body composition parameters, sarcopenia and high VAT have been reported as prognostic factors for mortality in sorafenib-treated patients with HCC [8,10,12,13]. Although muscle tissue generally contains only small amounts of adipose tissue, excessive accumulation of adipose tissue from abundant VAT may be considered a pathological feature termed myosteatosis. Myosteatosis can be easily detected in the mean density of muscle tissues using Hounsfield units (HUs) from CT. Unlike sarcopenia, which refers to a reduction in muscle mass, myosteatosis involves a decrease in muscle quality and is therefore considered to be an indicator of muscle function [14]. Recently, myosteatosis was shown to reflect low muscle function and contribute to prolonged muscle degradation, leading to unfavorable clinical outcomes in HCC [9,15]. However, no studies have investigated the prognostic value of these four body composition parameters for mortality in sorafenib-treated patients with HCC. Moreover, studies on the effect of myosteatosis on mortality in sorafenib-treated HCC patients are lacking.

In the present study, we retrospectively evaluated sarcopenia, visceral obesity using the visceral-to-subcutaneous adipose ratio (VSR), and myosteatosis using HUs and aimed to investigate the impact of the four body composition parameters on the survival of sorafenib-treated HCC patients.

## 2. Materials and Methods

### 2.1. Study Population

**Figure 1:**
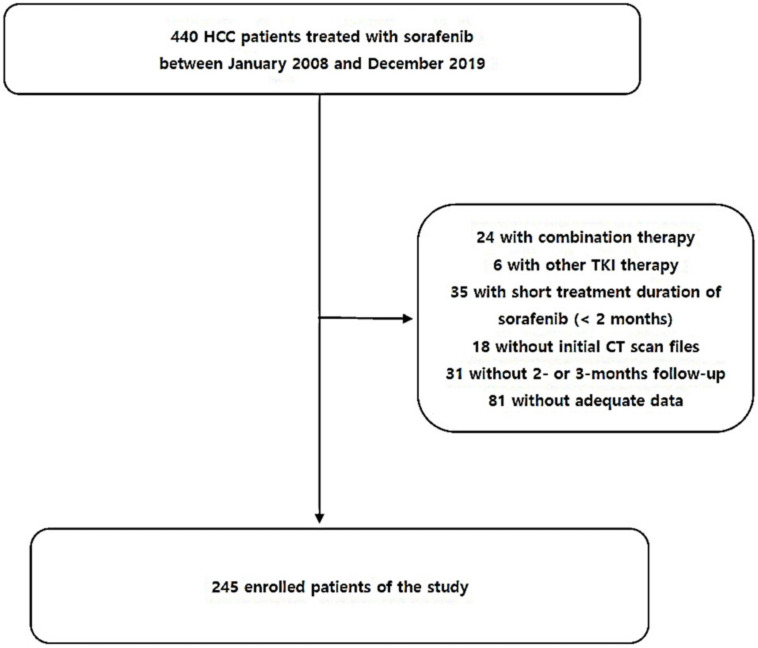
Study selection flowchart.

The medical records of patients were primarily collected by representatives from each institution (M.K.K., J.E.S., Y.R.L., and B.K.J.) and reviewed by other researchers at each hospital. A total of 440 HCC patients treated with sorafenib between January 2008 and December 2019 at four tertiary hospitals in Daegu, South Korea, were included. Enrolled patients were based on patients taking sorafenib orally at 400 mg twice daily, with a minimum dose of 400 mg per day. The exclusion criteria were as follows: combination therapy (*n* = 24); other systemic therapies including lenvatinib or regorafenib (*n* = 6); duration of sorafenib treatment < 2 months (*n* = 35); unavailability of initial CT scan files (*n* = 18); 2- or 3-month follow-up loss (*n* = 31) based on medical records; and inadequate data (*n* = 81). Ultimately, 245 patients were included in this study (Figure 1). The end date for mortality follow-up was December 2021.

This study was conducted in accordance with the ethical guidelines of the Helsinki Declaration of 1975, as revised in 2013. Owing to the retrospective nature of our study, the requirement for written informed consent from the patients was waived by the ethics committees of the four hospitals. The study protocol was approved by the Institutional Review Board of Yeungnam University Hospital (IRB No. 2021-08-029).

### 2.2. Assessment of Clinical Data

We retrospectively collected and reviewed the patients’ medical records covering anthropometric and epidemiological data, clinical characteristics, and laboratory and radiological data from each designated hospital. All data were based on values obtained at least 1 month prior to sorafenib administration. According to the guidelines of the European Association for the Study of the Liver, HCC was diagnosed based on typical radiological features using liver-specific dynamic CT or gadoxetic acid disodium–enhanced magnetic resonance imaging (MRI) [16]. Treatment response was assessed by experienced radiologists using the Response Evaluation Criteria in Solid Tumors (RECIST) 1.1 criteria of the first follow-up CT at 2 or 3 months [17]. An objective response was defined as a combination of complete and partial responses, and disease control was defined as an objective response with stable disease [17].

### 2.3. Assessment of Body Composition Data

Representatives from each institution (M.K.K., J.E.S., Y.R.L., and B.K.J.) evaluated the body composition parameters, including skeletal muscle, visceral and subcutaneous adipose tissue, and myosteatosis based on cross-sectional images of the third lumbar (L3) vertebra on abdominal CT. L3 body composition images were extracted using a picture archiving and communication system (Centricity, GE Healthcare, Chicago, IL, USA). The area of each body composition was calculated using well-validated, neural network-based, automated body composition analysis software (Automated Muscle and Adipose Tissue Composition Analysis (AutoMATiCA) https://gitlab.com/Michael_Paris/AutoMATiCA; assessed on 5 November 2021) using a brush tool [18].

**Figure 2:**
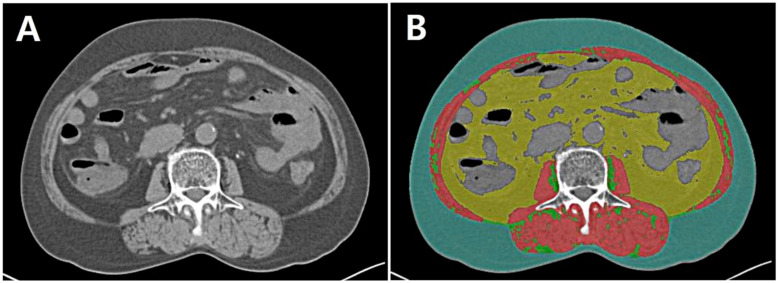
Cross-sectional abdominal computed tomography (CT) images at the L3 level. (**A**) Unenhanced (**B**) CT image with four body composition parameters applied using AutoMATiCA software (skeletal muscle area, red; visceral adipose tissue area, yellow; subcutaneous adipose tissue area, blue; intermuscular adipose tissue, green).

The skeletal muscle area (SMA) was quantified as the sum of the intra-abdominal muscles, based on tissue-specific standard HU thresholds of −29 to 150. The VAT area was quantified as the area of adipose tissue surrounding the internal skeletal muscles, excluding intra-abdominal organs such as the liver, kidneys, and intestine, based on standard HU thresholds of −150 to −50. The SAT area was quantified as the area of adipose tissue between the border of the skeletal muscles and the line of the abdominal skin, based on standard HU thresholds of −190 to −30. Inter/intra-muscular adipose tissue (IMAT) was quantified as fat deposition in the intra-abdominal muscle, based on standard HU thresholds of −190 to −30 (Figure 2).

The indices (cm^2^/m^2^) of the SMA, VAT, and SAT areas were defined as the skeletal muscle index (SMI), visceral adipose tissue index (VATI), and subcutaneous adipose tissue index (SATI), that is, the area of each body composition (cm^2^) divided by height squared (m^2^). Sarcopenia was defined as SMI < 50 cm^2^/m^2^ and <39 cm^2^/m^2^ in men and women, respectively [19]. Visceral obesity was defined as VSR > 1.33 for men and >0.93 for women [9]. Myosteatosis was defined as mean HUs of IMAT < 41 HUs and <33 HUs in patients with BMI < 25 kg/m^2^ and ≥25 kg/m^2^, respectively [20].

### 2.4. Study Outcomes

The primary and secondary endpoints were OS and PFS, respectively. OS was defined as the time from the first sorafenib administration to death or the last follow-up visit. PFS was defined as the time from the first sorafenib administration to radiological progression. Furthermore, we investigated the effect of body composition as an independent prognostic factor for mortality in sorafenib-treated patients with HCC.

### 2.5. Statistical Analysis

Continuous data are presented as medians with interquartile ranges (IQRs) after testing for normality. Categorical data are presented as numbers and percentages. Using the Kaplan–Meier method, the survival curve of each body composition parameter and additional predictive power according to the number of risk factors were implemented. The prognostic factors for OS and PFS in sorafenib-treated HCC patients were assessed using a Cox proportional regression model with the backward selection method. Statistical significance was set at *p* < 0.05. All statistical analyses were performed using R software (version 4.1.0; R Foundation for Statistical Computing, Vienna, Austria).

## 3. Results

### 3.1. Baseline Characteristics

The baseline characteristics of the enrolled patients are summarized in Table 1. In total, 245 sorafenib-treated HCC patients were included in this study. The median age of the patients was 67 years, and 211 (86.1%) were male. Of the patients, 66 (26.9%) and 75 (30.6%) had obesity and diabetes mellitus (DM), respectively.

Regarding tumor profiles, 149 (66.5%) patients had multiple tumor lesions, 105 (42.9%) had vessel invasion, and 122 (49.8%) had extrahepatic metastasis. The most common BCLC stage was C (86.2%), followed by B (13.8%). Of the patients, 231 (94.3%) and 14 (5.7%) were classified as CTP classes A and B, respectively. Regarding laboratory profiles, 124 (53.0%) and 111 (55.5%) patients had alpha-fetoprotein (AFP) levels > 200 ng/mL and protein induced by vitamin K absence or antagonist-II levels > 400 mAU/mL, respectively. After 2 or 3 months of follow-up, the number of patients who achieved complete response, partial response, stable disease, and progressive disease were 1 (0.4%), 9 (3.7%), 113 (46.1%), and 122 (49.8%), respectively. The objective response and disease control rates were 4.1% and 50.2%, respectively.

Regarding body composition analyses, the median SMI, VATI, HUs, and VSR were 49.4 cm^2^/m^2^, 42.7 cm^2^/m^2^, 53.7 HUs, and 1.1, respectively. The prevalences of sarcopenia, visceral obesity, and myosteatosis were 46.9%, 49.8%, and 9.0%, respectively. The median OS and PFS were 7.9 months (IQR, 4.1–15.3 months) and 4.8 months (IQR, 2.9–9.5 months), respectively.

### 3.2. Impact of Body Composition on Survival in Sorafenib-Treated HCC Patients

**Figure 3:**
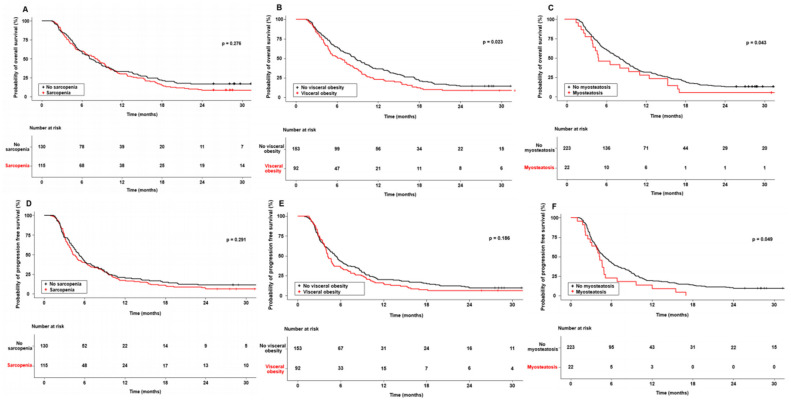
Survival plots (**A**–**F**) according to the body composition parameters in hepatocellular carcinoma patients treated with sorafenib; (**A**,**D**) survival plots according to the presence of sarcopenia; (**B**,**E**) survival plots according to the presence of visceral obesity; (**C**,**F**) survival plots according to the presence of myosteatosis.

Based on the body composition analysis, including sarcopenia, visceral obesity, and myosteatosis, we assessed the survival curves of OS and PFS in sorafenib-treated HCC patients (Figure 3).

Compared with patients without sarcopenia, those with sarcopenia were more likely to be male (76.2% vs. 97.4%, *p* < 0.001) and have a lower BMI (23.4 [21.4–25.8] vs. 22.4 [20.3–24.3] kg/m^2^, *p* = 0.023). Sarcopenia had no significant effect on OS (*p* = 0.276) or PFS (*p* = 0.291). The 6-, 12-, 18- and 24-month OS rates in the non-sarcopenic group were 60.0%, 30.0%, 15.4%, and 8.5%, respectively, and 59.1%, 33.0%, 20.9%, and 16.5% in the sarcopenic group, respectively. Compared with patients without visceral obesity, those with obesity were older (65.0 [57.0–75.0] vs. 71.0 [63.5–79.0] years, *p* < 0.001) and were more likely to be female (6.2% vs. 25.3%, *p* < 0.001). The presence of visceral obesity was associated with significantly poorer OS than the absence of visceral obesity (*p* = 0.023). The 6-, 12-, 18- and 24-month OS rates in the non-visceral obesity group were 64.7%, 36.6%, 21.6%, and 14.4%, respectively, and 51.1%, 22.8%, 12.0%, and 8.7% in the visceral obesity group, respectively. However, visceral obesity had no statistically significant effect on PFS (*p* = 0.186).

Compared with patients without myosteatosis, those with myosteatosis were older (67.0 [60.0–75.5] vs. 78.5 [65.0–82.0] years, *p* = 0.003) and had unfavorable survival outcomes for OS (*p* = 0.043) and PFS (*p* = 0.049). The 6-, 12-, 18- and 24-month OS rates in the non-myosteatosis group were 61.0%, 31.8%, 19.3%, and 13.0%, respectively, and 45.5%, 27.3%, 4.5%, and 4.5% in the myosteatosis group, respectively. Additionally, the 6-, 12-, 18- and 24-month PFS rates in the non-myosteatosis group were 42.6%, 19.3%, 13.9%, and 9.4%, respectively, and 22.7%, 9.1%, 4.6%, and 0% in the myosteatosis group, respectively.

### 3.3. Factors Associated with Survival in HCC Patients Treated with Sorafenib

We investigated the association between body composition parameters and survival in sorafenib-treated HCC patients, independent of classic risk factors (Table 2). In the Cox proportional hazard regression analysis, the presence of vessel invasion (hazard ratio (HR), 1.727; 95% confidence interval (CI), 1.258–2.371; *p* = 0.001), extrahepatic metastasis (HR, 1.401; 95% CI, 1.028–1.908; *p* = 0.033), and AFP level > 200 ng/mL (HR, 1.559; 95% CI, 1.105–2.201; *p* = 0.012) were independent tumor-related risk factors for OS. Furthermore, visceral obesity (HR, 1.478; 95% CI, 1.062–2.057; *p* = 0.021) and myosteatosis (HR, 1.814; 95% CI, 1.112–2.960; *p* = 0.017) were significant predictive factors for OS in patients treated with sorafenib.

In addition, the presence of vessel invasion (HR, 1.480; 95% CI, 1.104–1.982; *p* = 0.009), extrahepatic metastasis (HR, 1.456; 95% CI, 1.092–1.940; *p* = 0.011), and AFP level > 200 ng/mL (HR, 1.395; 95% CI, 1.036–1.879; *p* = 0.028) were independent tumor-related risk factors for PFS. Compared with the risk factors for OS, myosteatosis (HR, 1.732; 95% CI, 1.085–2.767; *p* = 0.021) was the only reliable body composition factor associated with PFS in those treated with sorafenib.

### 3.4. The Adequate Number of Significant Prognostic Factors for Predicting Survival in Sorafenib-Treated HCC Patients

**Figure 4:**
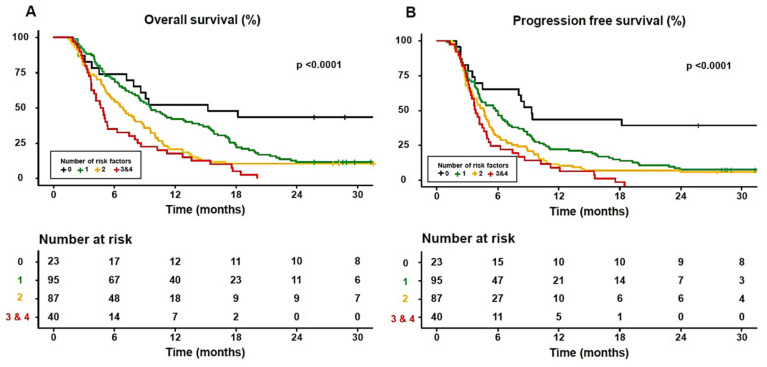
Survival plots according to the increased number of four risk factors including vessel invasion, extrahepatic metastasis, alpha-fetoprotein level > 200 ng/mL, and myosteatosis. (**A**) Overall survival and (**B**) progression-free survival.

Among the three body composition variables, only myosteatosis was a prognostic factor for two survival outcomes, OS and PFS, in sorafenib-treated HCC patients with HCC. Therefore, we performed a survival analysis to compare mortality according to four verified risk factors (vessel invasion, extrahepatic metastasis, AFP level > 200 ng/mL, and myosteatosis) using multivariate analysis. As the number of risk factors increased, the 1-year OS and PFS rates of the patients significantly decreased (no risk factors, 52.2% and 43.5%; one risk factor, 42.1% and 22.1%; two risk factors, 20.7% and 12.5%; three and four risk factors, 17.5% and 11.3%, respectively) (all *p* < 0.0001) (Figure 4).

**Figure 5:**
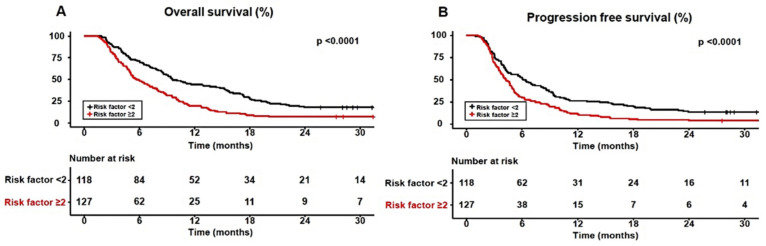
Survival plots between the high-risk group (≥2 risk factors) and low-risk group (<2 risk factor). (**A**) Overall survival and (**B**) progression-free survival.

To explore the additional predictive performance of risk factors in terms of survival, we classified the patients into two groups: a low-risk group with <2 risk factors and a high-risk group with ≥2 risk factors. Compared with the low-risk group, the high-risk group were younger (70.0 [62.0–79.0] vs. 65.0 [58.0–74.0] years, *p* = 0.004) and more likely to be female (18.9% vs. 8.5%, *p* = 0.003). The 6-, 12-, 18- and 24-month OS rates in the low-risk group were 71.2%, 44.1%, 28.0%, and 17.8%, respectively, and 48.8%, 19.7%, 8.6%, and 7.1% in the high-risk group, respectively. The 6-, 12-, 18- and 24-month PFS rates in the low-risk group were 52.5%, 26.3%, 20.3%, and 13.6%, respectively, and 29.9%,11.0%, 5.5%, and 3.9% in the high-risk group, respectively. Consequently, the high-risk group had a significantly worse survival prognosis than the low-risk group (all *p* < 0.0001) (Figure 5).

## 4. Discussion

Based on the analysis of the four body composition parameters, we found that visceral obesity was associated with unfavorable OS outcomes in sorafenib-treated HCC patients. Furthermore, we found that myosteatosis was associated with poor prognostic factors for survival in sorafenib-treated HCC patients, independent of traditional risk factors, including vascular invasion, extrahepatic metastasis, and AFP levels > 200 ng/mL. Using four shared risk factors for OS and PFS, we demonstrated that patients with more than two risk factors had worse prognosis than those without. Our results suggest the applicability of myosteatosis as a novel CT-based body composition prognostic factor in sorafenib-treated HCC patients.

Recent studies revealed the role of myosteatosis as a prognostic factor in Japanese patients with HCC [9,15,21]. However, evidence of an association between myosteatosis and mortality in sorafenib-treated HCC patients is lacking. Fujiwara et al. demonstrated that intramuscular fat deposition (i.e., myosteatosis) predicts mortality in a large-scale cohort of patients with HCC [9]. However, the proportion of enrolled patients with BCLC-C stage was approximately 4%, which differed from the 86.2% proportion in our study. Two studies have shown that myosteatosis is predictive of unfavorable outcomes after hepatectomy in patients with HCC [15,21]. In a meta-analysis, myosteatosis was a prognostic factor for poor OS in patients with HCC (HR: 1.88; 95% CI, 1.40–2.52; *p* < 0.0001) but was not evaluated for poor PFS [22]. We found that myosteatosis had an approximately two-fold HR for both OS and PFS. Another study demonstrated that myosteatosis is associated with the probability of predicting OR and treatment failure in patients with advanced HCC treated with immune hepatic artery infusion chemotherapy [23]. Contrary to previous studies, our study had practical considerations as a multicenter cohort study to determine the impact of myosteatosis on sorafenib-treated HCC patients, independent of traditional prognostic factors.

Compared with myosteatosis, visceral obesity is a relatively well-known prognostic factor for predicting mortality in HCC patients [9,12,13,15,24]. Some studies have revealed an association between visceral obesity and unfavorable clinical outcomes after hepatectomy in HCC patients [9,15,24]. However, studies on the effects of visceral obesity in sorafenib-treated HCC patients are controversial because of the discordance in the definition of visceral fat. Similar to our results, Nault et al. demonstrated that high visceral fat area was associated with worse OS in patients with advanced HCC treated with TKIs [12]. Saeki et al. demonstrated that the absence of muscle depletion with a high visceral fat area was a significant predictor of survival in sorafenib-treated patients with advanced HCC [13]. However, a Dutch study with more patients showed that a high visceral fat area was not associated with impaired survival, independent of known prognostic factors, in sorafenib-treated HCC patients [11]. Contrary to the results of previous studies, visceral obesity was identified as a sex-specific coefficient of variance (COV) of VSR in the current study. The rationale for applying visceral obesity using VSR and the pathophysiological mechanisms of myosteatosis and visceral obesity in patients with HCC are as follows.

Adipose tissue, a secretory organ that produces various adipokines and pro- and anti-inflammatory cytokines, is classified as VAT or SAT according to the distribution of fat in the abdomen [25,26,27]. Cytokine imbalance plays an important role in pathophysiological processes, including insulin resistance (IR), chronic inflammation, and alterations in glucose and lipid metabolism [25,26,27]. Previous studies have shown that cytokine production profiles differ between VAT and SAT; VAT produces pro-inflammatory cytokines, including interleukin 6 and tumor necrosis factor-alpha, whereas SAT secretes anti-inflammatory cytokines, including adiponectin [28,29]. Compared with the measurement of VAT alone, the measurement of VSR may be a method to adjust the conflicting cytokine balance.

Furthermore, surplus VAT is associated with immune dysfunction through the dysregulation of increased leptin and decreased adiponectin levels, the activation of immune cells, and alteration of cell-mediated immune responses [28]. Some studies have shown that leptin has oncogenic activity via activation of the PI3K/Akt pathway and signal transducer and activator of transcription (STAT) 3 and STAT5 in tumor cells, whereas adiponectin inhibits leptin-associated pathways [25,30]. These results suggest that visceral obesity may worsen the prognosis of patients with HCC.

Although the putative mechanism underlying myosteatosis in HCC is not fully understood, the association between myosteatosis and visceral obesity is a possible mechanism. Due to the limited expandability of adipose tissue, excessive fat accumulates in skeletal muscle. Myosteatosis is closely associated with chronic inflammation and prolonged IR, leading to mitochondrial dysfunction, myocellular apoptosis, and muscle degradation [31]. Altered myokines may affect the abnormal secretion of leptin and adiponectin via adipokine–myokine crosstalk, leading to increased oncogenic activity.

Sarcopenia may be a prognostic factor for TKI-treated HCC patients [8,10,32,33]. In a recent meta-analysis, TKI-treated patients with sarcopenia, including treatment with sorafenib or lenvatinib, had lower OS than those without, contrary to our results (HR, 2.24; 95% CI, 1.60–3.14, *p* < 0.001) [33]. Our views on the conflicting results regarding sarcopenia are as follows. A previous study revealed that adipose tissue regulation occurs in the initial stage of cancer-associated cachexia, prior to sarcopenia [34]. Sarcopenia is a prognostic factor for mortality in patients with end-stage liver disease such as decompensated cirrhosis, whereas adipopenia is related to mortality in patients with compensated cirrhosis [19,35]. Considering that sorafenib-treated HCC patients have preserved liver function and favorable performance status, they might have been placed in a favorable nutritional condition far from muscle depletion of the exacerbating catabolic state. Additionally, selection bias, discordance in the definition of sarcopenia using different COVs, and the application of different body composition parameters may have affected these conflicting findings. In another study that applied various body composition parameters, including visceral obesity and myosteatosis, sarcopenia was not associated with OS in sorafenib-treated HCC patients, which is consistent with our study [11].

Careful interpretation is required owing to the limitations of the present study. First, owing to its cross-sectional, retrospective Korean population cohort design, it is difficult to determine the causality between body composition parameters and clinical outcomes and to generalize the results to all HCC patients. Further large multinational prospective studies are required to confirm the causal impact of body composition parameters on HCC patients. Second, there is a possibility of selection bias owing to the enrolled patients who had been treated with sorafenib for >2 months and had an evaluated treatment response after 2–3 months. Although the effect of sarcopenia may have been underestimated because of the possibility that relatively favorable patients were included, we plan to investigate the effect of body composition parameters in future longitudinal studies. Third, Asian COVs for sarcopenia were not applied because they were satisfied by only two women. However, given that sorafenib-treated HCC patients have a favorable general condition with preserved liver function, myosteatosis and visceral obesity were preferentially associated with worse outcomes in the initial catabolic status prior to sarcopenia. Further large-scale longitudinal cohort studies using specific ethnic COVs as body composition parameters are required. Fourth, the software AutoMATiCA utilizes neural networks to analyze body composition. However, its accuracy in body composition segmentation may be affected by the patient’s general condition. In a comparison with conventional manual analysis software (Slice-O-Matic software (v.4.3; Tomovision, Montreal, QC, Canada)), AutoMATiCA showed a high Dice similarity coefficient (DSC) of over 0.9 for IMAT. However, in critically ill patients, including those in the intensive care unit and those with decompensated liver cirrhosis and ascites, IMAT showed lower DSC (0.55–0.69) than conventional software [18]. It is important to note that sorafenib-treated HCC patients are mostly compensated liver cirrhosis patients with good performance status, so it can be assumed that they will have good performance scans. Future studies are needed to improve the accuracy of automatic imaging analysis in larger cohorts. Fifth, due to the low prevalence of myosteatosis (9%), the statistical power of the parameter may be weak. However, in a meta-analysis, the prevalence of myosteatosis ranged from 11% to 85%. The authors acknowledge that the cut-points of myosteatosis and the severity of the patient can influence the variation [22]. Given the lack of consensus on the definition of myosteatosis in the field of oncology, further large-scale studies are warranted. Finally, we were unable to establish an association between myosteatosis and nutritional status due to the lack of results for prealbumin and transferrin in the study. This study found no correlation between myosteatosis and serum albumin levels (r_s_ by Spearman’s correlation = −0.08, *p* = 0.221). Future studies are needed to elucidate the association between laboratory tests reflecting nutritional status and body composition parameters in cancer patients.

## 5. Conclusions

Notwithstanding the aforementioned limitations, we are the first group to reveal the effect of myosteatosis on mortality in sorafenib-treated HCC patients using four body composition parameters. Thus, myosteatosis may be a novel prognostic CT-based radiological biomarker for previously established mortality-independent risk factors. Additionally, we have presented evidence for the establishment of a new prediction-based model using a combination of classic risk factors, including myosteatosis. Through external validation and molecular studies in a prospective study, a novel body composition-based prediction model for clinical outcomes and the discovery of putative molecular mechanisms of myosteatosis in patients with HCC is required.

## Figures and Tables

**Table 1:** Baseline characteristics of enrolled patients.

Variable	Enrolled Patients *n* = 245
Age, year	67.0 [61.0–78.0]
Men, *n* (%)	211 (86.1)
Body mass index, kg/m^2^	22.8 [20.9–25.3]
Etiology, *n* (%)	
HBV/HCV/Alcohol/other	154(62.8)/26 (10.5)/33 (13.4)/32(13.3)
Comorbidities, *n* (%)	
Obesity	66 (26.9)
Diabetes mellitus	75 (30.6)
Hypertension	75 (30.6)
**Tumor profiles**	
Single/multiple, *n* (%)	75 (33.5)/149 (66.5)
Largest diameter, mm	68.5 [33.0–110.0]
Vessel invasion, *n* (%)	105 (42.9)
Extrahepatic metastasis, *n* (%)	122 (49.8)
BCLC stage, *n* (%)	
B/C	34 (13.8)/211 (86.2)
CTP classification, *n* (%)	
A/B	231 (94.3)/14 (5.7)
**Laboratory profiles**	
Platelet count, ×10^9^/µL	137 [88.5–188.0]
Aspartate aminotransferase, IU/L	61.0 [40.0–105.0]
Alanine aminotransferase, IU/L	31.0 [20.0–50.0]
Serum albumin, g/dL	3.7 [3.4–4.1]
Prothrombin time, INR	1.1 [1.1–1.2]
Alpha-fetoprotein, >200 ng/mL, *n* (%)	124 (53.0)
PIVKA >400 mAU/mL, *n* (%)	111 (55.5)
**Response** at 2-/3-month follow-up, *n* (%)	
Complete response	1 (0.4)
Partial response	9 (3.7)
Stable disease	113 (46.1)
Progressive disease	122 (49.8)
Objective response rate, %	4.1
Disease control rate, %	50.2
**Body composition analyses based on CT**	
SMI, cm^2^/m^2^	49.4 [43.0–58.1]
VATI, cm^2^/m^2^	42.7 [23.5–62.7]
SATI., cm^2^/m^2^	36.7 [24.6–51.7]
HU	53.7 [48.3–56.6]
VSR	1.1 [0.8–1.5]
Sarcopenia, *n* (%)	115 (46.9)
Presence of visceral obesity, *n* (%)	92 (37.6)
Myosteatosis, *n* (%)	22 (9.0)
**Clinical outcomes**	
Overall survival, months	7.9 [4.1–15.3]
Progression-free survival, months	4.8 [2.9–9.5]

Values are expressed as the median (interquartile range (IQR)) or *n* (%). HBV, hepatitis B virus; HCV, hepatitis C virus; BCLC, Barcelona Clinic Liver Cancer; CTP, Child–Turcotte–Pugh; INR, international normalized ratio; PIVKA, prothrombin induced by vitamin K absence or antagonist-II; CT, computed tomography; SMI, skeletal muscle index; VATI, visceral adipose tissue index; SATI, subcutaneous adipose tissue index; HU, Hounsfield unit; VSR, visceral-to-subcutaneous fat ratio.

**Table 2:** Factors associated with survival outcome in patients with hepatocellular carcinoma treated with sorafenib.

Variables	Overall Survival				Progression-Free Survival			
	Univariate *p*-Value	Multivariate			Univariate *p*-Value	Multivariate		
		HR	95% CI	*p*-Value		HR	95% CI	*p*-Value
Age, years	0.677				0.729			
Male	0.354				0.657			
Obesity	0.534				0.909			
Diabetes mellitus	0.878				0.397			
Hypertension	0.928				0.777			
Tumor number, multiple vs. single	0.249				0.064			
Tumor size > 70 mm	0.001				0.029			
Vessel invasion	<0.001	1.727	1.258–2.371	0.001	0.011	1.480	1.104–1.982	0.009
Extrahepatic metastasis	0.081	1.401	1.028–1.908	0.033	0.009	1.456	1.092–1.940	0.011
CTP classification, B vs. A	0.045				0.336			
AFP > 200 ng/mL	0.003	1.559	1.105–2.201	0.012	0.093	1.395	1.036–1.879	0.028
PIVKA > 400 mAU/mL	0.272				0.992			
Sarcopenia, yes/no	0.276				0.291			
Visceral adiposity	0.023	1.478	1.062–2.057	0.021	0.186			
Myosteatosis	0.043	1.814	1.112–2.960	0.017	0.049	1.732	1.085–2.767	0.021

HR, hazard ratio; CI, confidence interval; CTP, Child–Turcotte–Pugh; AFP, alpha-fetoprotein; PIVKA, prothrombin induced by vitamin K absence or antagonist II.

## Data Availability

The data used to support the findings of this study are available from the corresponding author upon request.

## References

[1] Sung H., Ferlay J., Siegel R.L., Laversanne M., Soerjomataram I., Jemal A., Bray F. (2021). Global Cancer Statistics 2020: GLOBOCAN Estimates of Incidence and Mortality Worldwide for 36 Cancers in 185 Countries. CA Cancer J. Clin..

[2] Reig M., Forner A., Rimola J., Ferrer-Fabrega J., Burrel M., Garcia-Criado A., Kelley R.K., Galle P.R., Mazzaferro V., Salem R. (2022). BCLC strategy for prognosis prediction and treatment recommendation: The 2022 update. J. Hepatol..

[3] Llovet J.M., Kelley R.K., Villanueva A., Singal A.G., Pikarsky E., Roayaie S., Lencioni R., Koike K., Zucman-Rossi J., Finn R.S. (2021). Hepatocellular carcinoma. Nat. Rev. Dis. Primers.

[4] Iavarone M., Cabibbo G., Piscaglia F., Zavaglia C., Grieco A., Villa E., Camma C., Colombo M., SOFIA (SOraFenib Italian Assessment) study group (2011). Field-practice study of sorafenib therapy for hepatocellular carcinoma: A prospective multicenter study in Italy. Hepatology.

[5] Lencioni R., Kudo M., Ye S.L., Bronowicki J.P., Chen X.P., Dagher L., Furuse J., Geschwind J.F., de Guevara L.L., Papandreou C. (2014). GIDEON (Global Investigation of therapeutic DEcisions in hepatocellular carcinoma and Of its treatment with sorafeNib): Second interim analysis. Int. J. Clin. Pract..

[6] Cheng A.-L., Kang Y.-K., Chen Z., Tsao C.-J., Qin S., Kim J.S., Luo R., Feng J., Ye S., Yang T.-S. (2009). Efficacy and safety of sorafenib in patients in the Asia-Pacific region with advanced hepatocellular carcinoma: A phase III randomised, double-blind, placebo-controlled trial. Lancet Oncol..

[7] Llovet J.M., Ricci S., Mazzaferro V., Hilgard P., Gane E., Blanc J.-F., De Oliveira A.C., Santoro A., Raoul J.-L., Forner A. (2008). Sorafenib in advanced hepatocellular carcinoma. N. Engl. J. Med..

[8] Nishikawa H., Nishijima N., Enomoto H., Sakamoto A., Nasu A., Komekado H., Nishimura T., Kita R., Kimura T., Iijima H. (2017). Prognostic significance of sarcopenia in patients with hepatocellular carcinoma undergoing sorafenib therapy. Oncol. Lett..

[9] Fujiwara N., Nakagawa H., Kudo Y., Tateishi R., Taguri M., Watadani T., Nakagomi R., Kondo M., Nakatsuka T., Minami T. (2015). Sarcopenia, intramuscular fat deposition, and visceral adiposity independently predict the outcomes of hepatocellular carcinoma. J. Hepatol..

[10] Imai K., Takai K., Miwa T., Taguchi D., Hanai T., Suetsugu A., Shiraki M., Shimizu M. (2019). Rapid Depletions of Subcutaneous Fat Mass and Skeletal Muscle Mass Predict Worse Survival in Patients with Hepatocellular Carcinoma Treated with Sorafenib. Cancers.

[11] Labeur T.A., van Vugt J.L.A., Ten Cate D.W.G., Takkenberg R.B., JNM I.J., Groot Koerkamp B., de Man R.A., van Delden O.M., Eskens F., Klumpen H.J. (2019). Body Composition Is an Independent Predictor of Outcome in Patients with Hepatocellular Carcinoma Treated with Sorafenib. Liver Cancer.

[12] Nault J.C., Pigneur F., Nelson A.C., Costentin C., Tselikas L., Katsahian S., Diao G., Laurent A., Mallat A., Duvoux C. (2015). Visceral fat area predicts survival in patients with advanced hepatocellular carcinoma treated with tyrosine kinase inhibitors. Dig. Liver Dis..

[13] Saeki I., Yamasaki T., Maeda M., Kawano R., Hisanaga T., Iwamoto T., Matsumoto T., Hidaka I., Ishikawa T., Takami T. (2018). No Muscle Depletion with High Visceral Fat as a Novel Beneficial Biomarker of Sorafenib for Hepatocellular Carcinoma. Liver Cancer.

[14] Meister F.A., Lurje G., Verhoeven S., Wiltberger G., Heij L., Liu W.-J., Jiang D., Bruners P., Lang S.A., Ulmer T.F. (2022). The role of sarcopenia and myosteatosis in short-and long-term outcomes following curative-intent surgery for hepatocellular carcinoma in a European cohort. Cancers.

[15] Hamaguchi Y., Kaido T., Okumura S., Kobayashi A., Shirai H., Yao S., Yagi S., Kamo N., Seo S., Taura K. (2019). Preoperative Visceral Adiposity and Muscularity Predict Poor Outcomes after Hepatectomy for Hepatocellular Carcinoma. Liver Cancer.

[16] Liver E.A.F.T.S.O.T. (2018). EASL clinical practice guidelines: Management of hepatocellular carcinoma. J. Hepatol..

[17] Therasse P., Arbuck S.G., Eisenhauer E.A., Wanders J., Kaplan R.S., Rubinstein L., Verweij J., Van Glabbeke M., van Oosterom A.T., Christian M.C. (2000). New guidelines to evaluate the response to treatment in solid tumors. J. Natl. Cancer Inst..

[18] Paris M.T., Tandon P., Heyland D.K., Furberg H., Premji T., Low G., Mourtzakis M. (2020). Automated body composition analysis of clinically acquired computed tomography scans using neural networks. Clin. Nutr..

[19] Carey E.J., Lai J.C., Wang C.W., Dasarathy S., Lobach I., Montano-Loza A.J., Dunn M.A., Fitness L.E., Exercise in Liver Transplantation C. (2017). A multicenter study to define sarcopenia in patients with end-stage liver disease. Liver Transpl..

[20] Martin L., Birdsell L., MacDonald N., Reiman T., Clandinin M.T., McCargar L.J., Murphy R., Ghosh S., Sawyer M.B., Baracos V.E. (2013). Cancer cachexia in the age of obesity: Skeletal muscle depletion is a powerful prognostic factor, independent of body mass index. J. Clin. Oncol..

[21] Kaibori M., Ishizaki M., Iida H., Matsui K., Sakaguchi T., Inoue K., Mizuta T., Ide Y., Iwasaka J., Kimura Y. (2015). Effect of Intramuscular Adipose Tissue Content on Prognosis in Patients Undergoing Hepatocellular Carcinoma Resection. J. Gastrointest. Surg..

[22] Aleixo G.F.P., Shachar S.S., Nyrop K.A., Muss H.B., Malpica L., Williams G.R. (2020). Myosteatosis and prognosis in cancer: Systematic review and meta-analysis. Crit. Rev. Oncol. Hematol..

[23] Yi X., Fu Y., Long Q., Zhao Y., Li S., Zhou C., Lin H., Liu X., Liu C., Chen C. (2022). Myosteatosis can Predict Unfavorable Outcomes in Advanced Hepatocellular Carcinoma Patients Treated With Hepatic Artery Infusion Chemotherapy and Anti-PD-1 Immunotherapy. Front. Oncol..

[24] Jang H.Y., Choi G.H., Hwang S.H., Jang E.S., Kim J.W., Ahn J.M., Choi Y., Cho J.Y., Han H.S., Lee J. (2021). Sarcopenia and visceral adiposity predict poor overall survival in hepatocellular carcinoma patients after curative hepatic resection. Transl. Cancer Res..

[25] Dalamaga M. (2013). Interplay of adipokines and myokines in cancer pathophysiology: Emerging therapeutic implications. World J. Exp. Med..

[26] Park J., Morley T.S., Kim M., Clegg D.J., Scherer P.E. (2014). Obesity and cancer—Mechanisms underlying tumour progression and recurrence. Nat. Rev. Endocrinol..

[27] Pedersen B.K., Febbraio M.A. (2012). Muscles, exercise and obesity: Skeletal muscle as a secretory organ. Nat. Rev. Endocrinol..

[28] Ghigliotti G., Barisione C., Garibaldi S., Fabbi P., Brunelli C., Spallarossa P., Altieri P., Rosa G., Spinella G., Palombo D. (2014). Adipose tissue immune response: Novel triggers and consequences for chronic inflammatory conditions. Inflammation.

[29] Marra F., Bertolani C. (2009). Adipokines in liver diseases. Hepatology.

[30] Vansaun M.N. (2013). Molecular pathways: Adiponectin and leptin signaling in cancer. Clin. Cancer Res..

[31] Laurens C., Moro C. (2016). Intramyocellular fat storage in metabolic diseases. Horm. Mol. Biol. Clin. Investig..

[32] Hiraoka A., Hirooka M., Koizumi Y., Izumoto H., Ueki H., Kaneto M., Kitahata S., Aibiki T., Tomida H., Miyamoto Y. (2017). Muscle volume loss as a prognostic marker in hepatocellular carcinoma patients treated with sorafenib. Hepatol. Res..

[33] March C., Omari J., Thormann M., Pech M., Wienke A., Surov A. (2022). Prevalence and role of low skeletal muscle mass (LSMM) in hepatocellular carcinoma. A systematic review and meta-analysis. Clin. Nutr. ESPEN.

[34] Petruzzelli M., Schweiger M., Schreiber R., Campos-Olivas R., Tsoli M., Allen J., Swarbrick M., Rose-John S., Rincon M., Robertson G. (2014). A switch from white to brown fat increases energy expenditure in cancer-associated cachexia. Cell Metab..

[35] Feng H., Wang X., Mao L., Yu Z., Cui B., Lin L., Hui Y., Zhao X., Xu X., Fan X. (2021). Relationship between sarcopenia/myosteatosis and frailty in hospitalized patients with cirrhosis: A sex-stratified analysis. Ther. Adv. Chronic Dis..

